# Variable Light Condition Improves Root Distribution Shallowness and P Uptake of Soybean in Maize/Soybean Relay Strip Intercropping System

**DOI:** 10.3390/plants9091204

**Published:** 2020-09-15

**Authors:** Li Wang, Tao Zhou, Bin Cheng, Yongli Du, Sisi Qin, Yang Gao, Mei Xu, Junji Lu, Ting Liu, Shuxian Li, Weiguo Liu, Wenyu Yang

**Affiliations:** 1 College of Agronomy, Sichuan Agricultural University, Chengdu 611130, Sichuan, China; S20176512@stu.sicau.edu.cn (L.W.); 2018201013@stu.sicau.edu.cn (B.C.); S20176538@stu.sicau.edu.cn (S.Q.); 2019101006@stu.sicau.edu.cn (Y.G.); 2018301013@stu.sicau.edu.cn (M.X.); 2019301099@stu.sicau.edu.cn (J.L.); lishuxian1@yeah.net (S.L.); 2 College of pharmacy, Chengdu University of Traditional Chinese Medicine, Chengdu 611137, Sichuan, China; zhoutao@cdutcm.edu.cn; 3 Institute of Sorghum Potato, Yibin Academy of Agricultural Sciences, Yibin 644000, Sichuan, China; duyongli123@yeah.net; 4 Agricultural and Rural Bureau of Sishui County, Jining 273200, Shandong, China; liuting727@yeah.net

**Keywords:** inter-cropping, light, auxin, root distribution, soybean, phosphorus

## Abstract

In this study, soybean root distribution in an inter-cropping system was influenced by various environmental and biotic cues. However, it is still unknown how root development and distribution in inter-cropping responds to aboveground light conditions. Herein, soybeans were inter- and monocropped with P (phosphorus) treatments of 0 and 20 kg P ha yr^−1^ (P0 and P20, respectively) in field experiment over 4 years. In 2019, a pot experiment was conducted as the supplement to the field experiment. Shade from sowing to V5 (Five trifoliolates unroll) and light (SL) was used to imitate the light condition of soybeans in a relay trip inter-cropping system, while light then shade from V5 to maturity (LS) was used to imitate the light condition of soybeans when monocropped. Compared to monocropping, P uptake and root distribution in the upper 0–15 cm soil layer increased when inter-cropped. Inter-cropped soybeans suffered serious shade by maize during a common-growth period, which resulted in the inhibition of primary root growth and a modified auxin synthesis center and response. During the solo-existing period, plant photosynthetic capacity and sucrose accumulation increased under ameliorated light in SL (shade-light). Increased light during the reproductive stage significantly decreased leaf P concentration in SL under both P-sufficient and P-deficient conditions. Transcripts of a P starvation response gene (*GmPHR25*) in leaves and genes (*GmEXPB2*) involved in root growth were upregulated by ameliorated light during the reproductive stage. Furthermore, during the reproductive stage, more light interception increased the auxin concentration and expression of *GmYUCCA14* (encoding the auxin synthesis) and *GmTIR1C* (auxin receptor) in roots. Across the field and pot experiments, increased lateral root growth and shallower root distribution were associated with inhibited primary root growth during the seedling stage and ameliorated light conditions in the reproductive stage. Consequently, this improved topsoil foraging and P uptake of inter-cropped soybeans. It is suggested that the various light conditions (shade-light) mediating leaf P status and sucrose transport can regulate auxin synthesis and respond to root formation and distribution.

## 1. Introduction

Phosphorus (P) is a critical element for plant growth and development, but its availability in soil is often limited [1,2]. In order to improve soil fertility and obtain a high crop yield, a large amount of P fertilizer has been put into farmland ecosystems. However, the excessive application of P fertilizer not only causes a waste of resources but destroys ecological environments through surface water runoff and eutrophication. In addition, P is a non-renewable mineral resource and studies have shown that half of known reserves will be consumed from 2040 to 2060 [3]. It is therefore important to enhance the use of accumulated P in soil by exploiting the biological potential of plants in cropping systems [4].

Inter-cropping is a farming practice that involves two or more crop species growing together or coexisting for a time. It is widely practiced worldwide and provides opportunities for the sustainable intensification of agriculture, as it offers a greater yield per unit land and a more efficient use of fertilizer than monocropping [5,6,7,8,9]. Inter-cropping is an internationally recognized efficient cropping system in terms of fertilizer resource utilization. Cereal/legume mixtures can explore various P resources and achieve higher P uptake than monocropping [10,11]. It is generally believed that the P uptake advantage of inter-cropping mainly comes from the following aspects: (1) under low P stress, legume root can release protons/hydroxyls, carboxylates, and enzymes to activate insoluble P in soil, and the P which is mobilized via legumes may be made available for cereal [12,13]. (2) Due to contrasting root architecture, spatial complementarity between cereals and legumes leads to the exploitation of P pools in different soil horizons [14]. (3) Indirect interspecific P facilitation in the rhizosphere can occur as a consequence of shifts in microbial biomass, community structure, and activity that contributes to increased P uptake [15,16]. (4) Compensation effects enable late-growing crops to obtain more resources after harvesting former crops (including the residual underground nutrients of the former crop and the better light environment), which is beneficial to nutrients uptake [17,18]. Researches on the advantages of nutrient absorption in inter-cropping mainly focused on interspecific interaction, while less attention paid to understand how light aboveground influences root growth, and subsequently P uptake [19,20,21,22]. Our previous study found that the nutrient acquisition and yield advantages of inter-cropped soybeans were mainly regulated by the aboveground light condition rather than interspecific root interactions in maize-soybean relay strip inter-cropping systems, especially in the area with low radiation [18,23].

It has been reported that root development and morphology are directly or indirectly regulated by aboveground light, such as primary root elongation, lateral root formation, nutrient absorption, etc. [24,25,26]. The light signal is mainly transmitted to the root in one of the following three ways: (i) transfer of mobile signaling messengers, (ii) roots that sense light directly, and (iii) light channeling through the stem [27]. Among these, the sucrose export from the shoot is a critical signal linking shoot light capture and root growth [28,29]. Moreover, it has been observed that sucrose plays an important role in the systemic control of plant P starvation responses [30]. In addition, the phytohormone auxin is an iconic long-distance signaling molecule that influences root development via polar transport [31]. Lateral root growth makes a considerable contribution to the spatial configuration of the root system in the soil and, subsequently, determines the ability of a plant to absorb nutrients in soil, such as P. Auxin. Its local accumulation in root pericycle cells are a necessary signal to prespecify these cells into lateral root founder cells [32]. Previous studies have proven that an increase in auxin levels and signaling is always accompanied by lateral root emergence, which increases the sufficient acquisition of lateral root founder cell identity [32].

Light can affect auxin at several level, i.e., its synthesis, transport, and signaling. Young tissues are the main source of auxin and are subsequently transported to the root, where it regulates root development, such as lateral root formation, that implicates auxin as a crucial integrator of light signal and root growth [33,34]. The transport of light-induced auxin from shoot to root is related to the E3 ubiquitin ligase (COP1), which acts as the main inhibitor of photomorphogenesis through the degradation of target proteins related to photoreaction [35]. In the dark, COP1 can mediate the transcriptional inhibition of PIN in the shoot by targeting the light morphology-inducing molecule HY5. However, the function of COP1 is inhibited under light, which can promote the transport of auxin to the root [36,37]. In the dark, the PIN1 and PIN2 proteins are located in the vacuoles of root cells, which could not perform a transport function, thus inhibiting the growth of taproot elongation. Under the light condition, PIN protein is located on the plasma membrane of root cells and performs a auxin transport function [38].

In the maize-soybean relay strip inter-cropping system, soybean experiences two very different periods: the common-growth and solo-existing periods. The growth of soybean during inter-cropping is inhibited by shade caused by maize during the common-growth period, but the inter-cropped soybean transfers to the full sunlight after the harvest of maize, and the ameliorated light condition substantially contributes in promoting soybean growth [6,18,39,40,41,42]. The ameliorated light condition during soybean’s reproductive stage of in inter-cropping increases the total root length and surface, which is associated with lower P concentrations in leaves and greater sucrose allocation [18]. However, root growth stimulation by increased light capture might not be controlled solely by increased sucrose allocation to roots. Instead, other systemic signals can be involved in this regulation process. For instance, auxin plays a major role in plant root formation [31,33,43,44]. Moreover, to date, it is not clear how the light-mediated auxin pathway regulates the growth and distribution in the soil of soybean roots, especially under variable light conditions.

In the present study, we conducted a long-term field experiment with a maize-soybean relay strip in an inter-cropped system, as well as a pot experiment to simulate the light environment of soybean, to investigate how the variable light condition of inter-cropped soybeans involved in regulating root growth and distribution in soil profiles, and the capacity of P uptake. We specifically examined whether the variable light condition and the auxin pathway were associated with the shallow distributed root of inter-cropped soybeans. Our hypothesis was that the formation of shallower distributed root systems might occur via a variable light condition, which (i) results in increased root auxin concentration or (ii) mediated shoot P status to regulate auxin response. This eventually shaped the P-efficient root architecture. Therefore, the aboveground light affected plant development and was taken as the entry point of this work. We studied soybeans in the relay strip inter-cropped system to investigate how the light condition mediates shoot P status and auxin pathway root growth and distribution in soil. Ultimately, it was revealed that, from the aspect of above-underground interactions, the mechanisms of nutrients efficiently uptake in the inter-cropping system.

## 2. Results

### 2.1. Yield and P Uptake Capability

**Figure 1:**
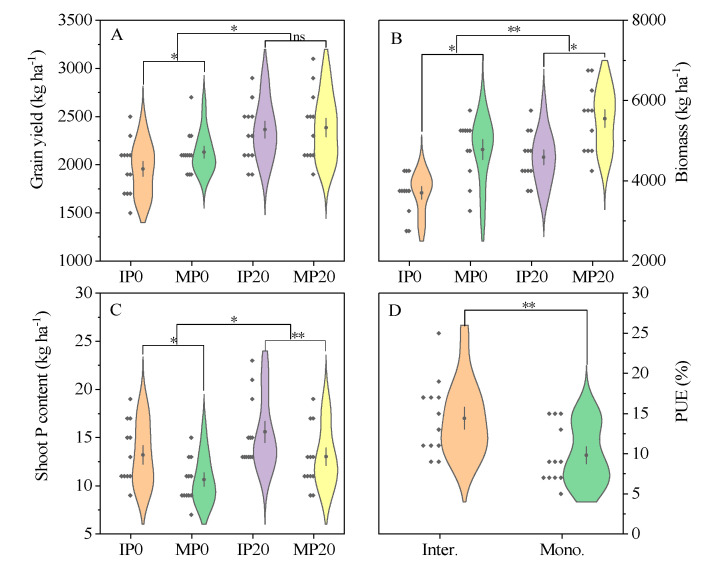
The effect of P (Phosphorus) application and cropping system on soybean (**A**) grain yield, (**B**) biomass, (**C**) shoot P content, and (**D**) PUE (P fertilizer-use efficiency) in the field experiment from 2016 to 2019. IP0: soybean was grown in inter-cropping without P application; MP0: soybean was grown in monocropping without P application; IP20: soybean was grown in inter-cropping with 20 kg P ha^−1^ application; MP20: soybean was grown in monocropping with 20 kg P ha^−1^ application; ** means the difference between treatment was significant (*p* ≤ 0.01) by “*t*” test; * means the difference between treatment was significant (*p* ≤ 0.05) by “*t*” test; ns means no difference.

The results of a 4-year orientation experiment showed that soybean grain yield of conventional P supply treatment (P20) was significantly (*p* ≤ 0.05) higher than that of zero-P supply treatment (P0), irrespective of cropping system (Figure 1A). In the P0 treatment, the grain yield in monocropping was significantly (*p* ≤ 0.05) higher than inter-cropping, while no significant (*p* > 0.05) difference was observed between them in the P20 treatment across all 4 years (Figure 1A).

Regardless of cropping system, P fertilizer significantly (*p* ≤ 0.01) increased the shoot dry weight (Figure 1B). The biomass accumulation in monocropping was significantly (*p* ≤ 0.05) higher than inter-cropping (Figure 1B). Nevertheless, the shoot growth rate of inter-cropped soybeans was greater than monocropped soybeans from V5 to R3 stages. The gap between them was decreased in the reproduction stages (Table S1). We did not observe any significant (*p* > 0.05) soybean growth rate when P fertilizer was applied.

Compared with zero-P application, conventional P application significantly (*p* ≤ 0.05) increased P accumulation irrespective of the cropping system (Figure 1C). The shoot P uptake and apparent P fertilizer efficiency (PUE) of inter-cropped soybeans was significantly (*p* ≤ 0.01) higher than monocropped soybeans (Figure 1C,D). From 2016 to 2019, the average PUE of the inter-cropping system was 14.3%, which was 58.9% higher than monocropping (Figure 1D).

### 2.2. Root Distribution and Morphological Traits

**Figure 2:**
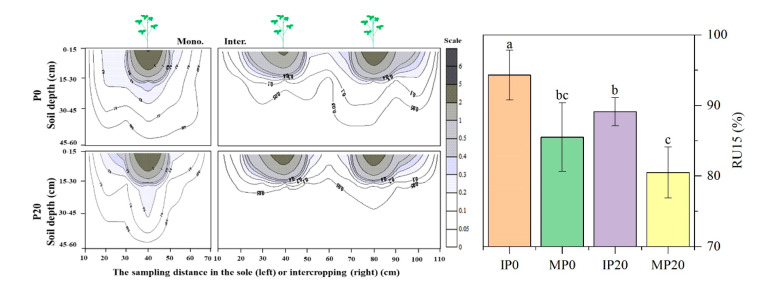
The effect of P application and cropping system on a two-dimensional distribution of soybean roots [g (5 dm^−3^)] in the top 60 cm of the soil profile at the R3 stage in the field experiment. Plants were grown in the inter-cropping (Inter.) and monocropping (Mono.) systems with two P application rates (P0 = 0 P application; P20 = 20 kg P ha^−1^ application). Values represent the mean of three replicates ± SE. Different letters on the columns are used to indicate means that differ significantly by LSD (*p* ≤ 0.05).

In the field experiment, the cropping system changed the distribution of soybean root in the soil. More than 90% of the roots in the inter-cropping system were distributed in the upper 0–15 cm soil layer; the deepest was about 45 cm (Figure 2). However, most of the roots in the monocropping system were distributed in the 0–30 cm layer; the deepest was about 55 cm (Figure 2). The RU15 (the proportion of roots distributed in the upper 15 cm soil layer) of inter-cropped soybeans was significantly (*p* ≤ 0.05) higher than monocropped soybeans, irrespective of the cropping system. Compared to P20 treatment, roots distribution was shallower in P0. In the horizontal direction, inter-cropped soybeans allocated more roots to the wide row side, while reduced allocation to the narrow row. For monocropped soybean, the root systems on both sides of the soybean were almost symmetrically distributed (Figure 2).

**Figure 3:**
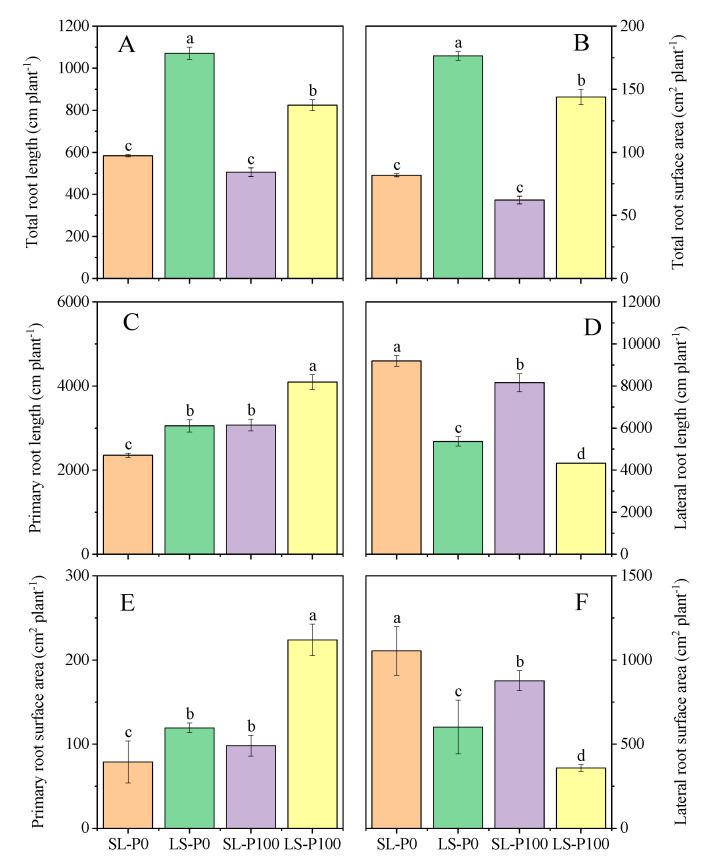
The effect of P application and light condition on (**A**) total root length, (**B**) total root surface at the V5 stage (Five trifoliolates unroll), the (**C**) primary root length, (**D**) lateral root length, (**E**) primary root surface, and (**F**) lateral root surface at the R3 stage (Pods are about 5 mm long on one of the four uppermost nodes on the main stem with a fully developed leaf) in the pot experiment. Plants were grown in two light conditions (SL: shade from sowing to the V5 stage and from V5 to R8 (95% of pods reach mature pod color) under natural light condition; LS: from sowing to the V5 stage under natural light condition and shade from V5 to R8) and two P application treatments (P0 = without P application; P100 = 100 mg P kg^−1^soil application). Values represent the mean of three replicates ± SE. Different letters on the columns are used to indicate means that differ significantly by LSD (*p* ≤ 0.05).

In the pot experiment, the total root length and root surface area of soybeans in LS treatments were significantly (*p* ≤ 0.05) higher than in SL treatments at the V5 stage, irrespective of P application (Figure 3A,B), while the total root length and surface area in SL treatment were significantly (*p* ≤ 0.05) greater than LS at the R3 stage. The effects of light intensity on root morphological traits were more pronounced when plants were grown without P application rather than soybeans supplied with sufficient P. Additionally, the increased total root length and surface area of soybeans in SL treatment were caused by increased length of lateral root, as the primary root length and surface area of soybeans in LS treatment were still significantly (*p* ≤ 0.05) greater than SL treatment at the R3 stage (Figure 3C–F).

**Figure 4:**
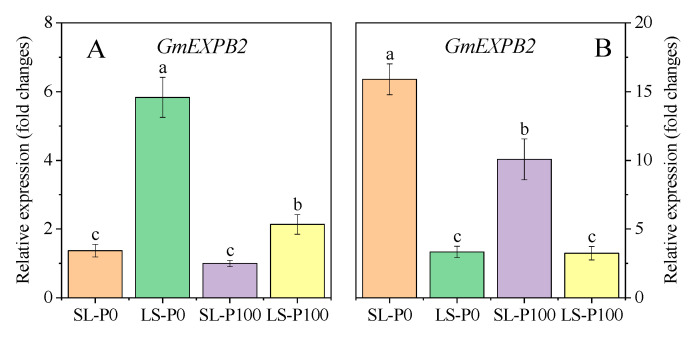
The effect of P application and light condition on the expression of gene *GmEXPB2* in roots ((**A**): V5 stage, (**B**): R3 stage) in the pot experiment. Plants were grown in two light conditions (SL: shade from sowing to the V5 stage and from V5 to R8 under natural light condition; LS: from sowing to the V5 stage under natural light condition and shade from V5 to R8) and two P application treatments (P0 = without P application; P100 = 100 mg P kg^−1^soil application). Values represent the mean of three replicates ± SE. Different letters on the columns are used to indicate means that differ significantly by LSD (*p* ≤ 0.05). For *GmEXPB2* expression, data are expressed as relative values based on the expression of *GmEXPB2* in roots of soybean grown under the treatment of SL-P100 at V5 stage referenced as 1.0.

The expression of *GmEXPB2*, which was associated with root growth and P uptake, was determined by P supply and light condition. More light capture and low-P supply increased the expression of *GmEXPB2* in the V5 and R3 stages (Figure 4).

### 2.3. Photosynthetic Efficiency and Sucrose Accumulation

**Figure 5:**
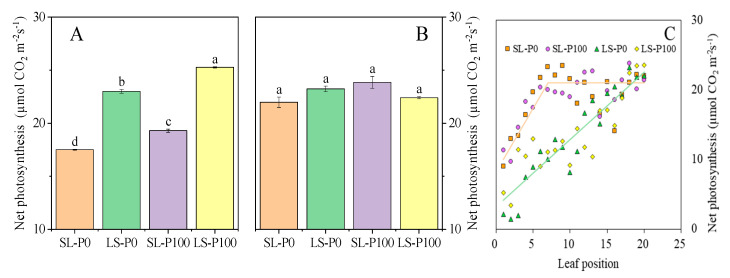
The effect of P application and light condition on net photosynthesis of the youngest fully expanded leaf ((**A**): V5 stage, (**B**): R3 stage) and (**C**) the whole plant Pn (Photosynthesis) was measured from the leaves situated lowest on the main stem to the highest (counted for 20 trifoliate leaves in soybean at R3 stage) in the pot experiment. Plants were grown in two light conditions (SL: shade from sowing to the V5 stage and from V5 to R8 under natural light condition; LS: from sowing to the V5 stage under natural light condition and shade from V5 to R8) and two P application treatments (P0 = without P application; P100 = 100 mg P kg^−1^soil application). Values represent the mean of three replicates ± SE. Different letters on the columns are used to indicate means that differ significantly by LSD (*p* ≤ 0.05).

At the V5 stage, net photosynthesis (Pn) of the youngest fully expanded leaf supplied with adequate P was significantly (*p* ≤ 0.05) higher than the zero-P supply. Increasing light intensity increased the Pn (Figure 5A). However, neither the light condition nor P application had a significant (*p* > 0.05) effect on the Pn of the youngest fully expanded leaf at the R3 stage (Figure 5B). The Pn of all soybean leaves from the oldest to the youngest were measured. We found that the Pn of old leaves in the LS treatment sharply decreased, while all leaves in the SL treatment maintained a relatively high level of Pn (Figure 5C).

**Figure 6:**
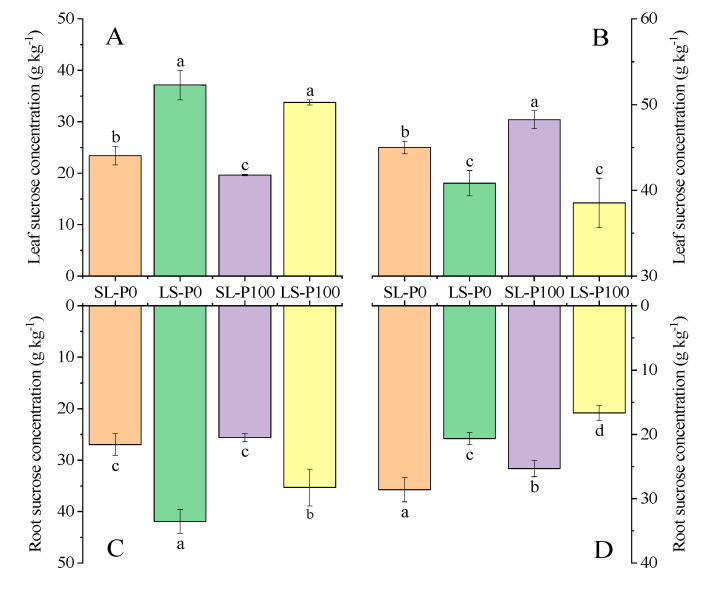
The effect of P application and light condition on the leaf sucrose concentration ((**A**): V5 stage, (**B**): R3 stage), root sucrose concentration ((**C**): V5 stage, (**D**): R3 stage) in the field experiment. Plants were grown in two light conditions (SL: shade from sowing to the V5 stage and from V5 to R8 under natural light condition; LS: from sowing to the V5 stage under natural light condition and shade from V5 to R8) and two P application treatments (P0 = without P application; P100 = 100 mg P kg^−1^soil application). Values represent the mean of three replicates ± SE. Different letters on the columns are used to indicate means that differ significantly by LSD (*p* ≤ 0.05).

At the V5 stage, the sucrose concentration in roots and leaves in LS were significantly (*p* ≤ 0.05) higher than SL treatments, especially in roots (Figure 6A,C). On the contrary, sucrose concentration in roots and leaves in SL were significantly (*p* ≤ 0.05) greater than LS at the R3 stage. Leaf sucrose concentration increased with P supply, while the root sucrose concentration in the P0 treatment was significantly (*p* ≤ 0.05) higher than in the P100 treatment (Figure 6B,D).

### 2.4. P Status in Leaf

**Figure 7:**
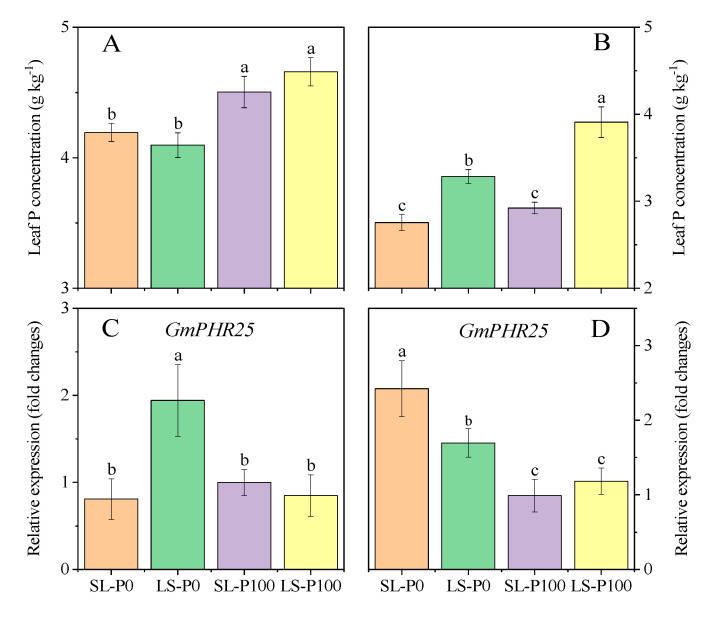
The effect of P application and light condition on the leaf P concentration ((**A**): V5 stage, (**B**): R3 stage), and the expression of gene *GmPHR25* in leaf ((**C**): V5 stage, (**D**): R3 stage) in the pot experiment. Plants were grown in two light conditions (SL: shade from sowing to the V5 stage and from V5 to R8 under natural light condition; LS: from sowing to the V5 stage under natural light condition and shade from V5 to R8) and two P application treatments (P0 = without P application; P100 = 100 mg P kg^−1^soil application). Values represent the mean of three replicates ± SE. Different letters on the columns are used to indicate means that differ significantly by LSD (*p* ≤ 0.05). For *GmPHR25* expression, data are expressed as relative values based on the expression of *GmPHR25* in roots of soybean grown under the treatment of SL-P100 at the V5 stage, referenced as 1.0.

In the P100 treatment, P concentration in leaves was significantly (*p* ≤ 0.05) higher than in P0 treatment, but the light condition had no significant (*p* > 0.05) effect on the leaf P concentration at the V5 stage (Figure 7A). Meanwhile, it was observed that *GmPHR25*, which was involved in the P starvation response, was highly expressed in the LS treatment compared with SL (Figure 7C). Both increasing light capture and low P condition significantly (*p* ≤ 0.05) decreased the leaf P concentration at the R3 stage (Figure 7B). The expression of *GmPHR25* in SL was significantly (*p* ≤ 0.05) higher compared to LS (Figure 7D). Whether it was at the V5 or R3 stage, the expressions of *GmPHR25* between two light conditions showed significant (*p* ≤ 0.05) difference only under P-deficient treatments, but no significant (*p* > 0.05) difference under P-sufficient treatments (Figure 7C,D).

### 2.5. IAA Concentration and Expression of Auxin-Related Gene

**Figure 8:**
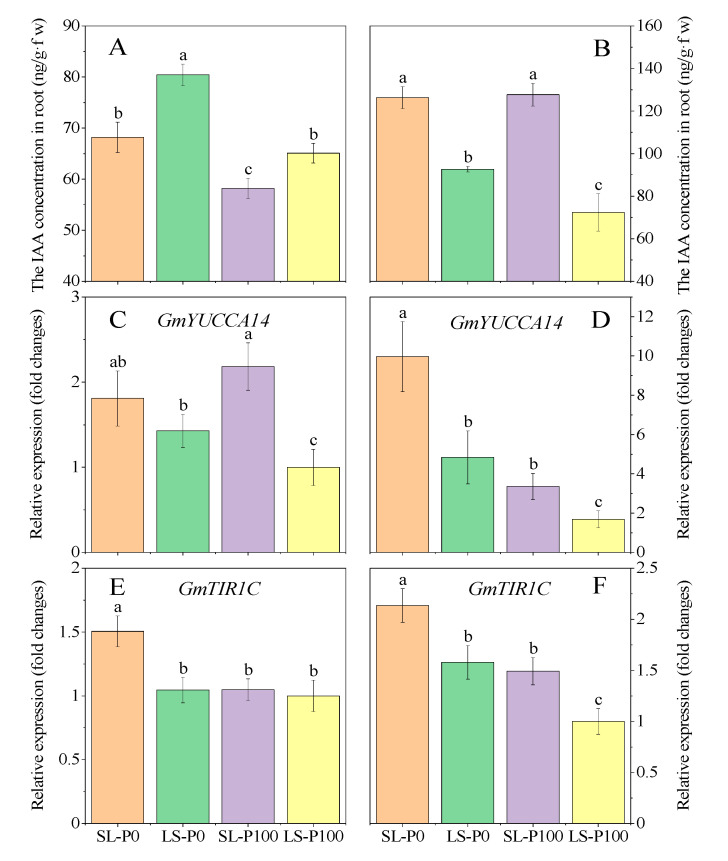
The effect of P application and light condition on the IAA concentration ((**A**): V5 stage, (**B**): R3 stage), and the expression of gene *GmYUCCA14* ((**C**): V5 stage, (**D**): R3 stage), *GmTIR1C* ((**E**): V5 stage, (**F**): R3 stage) in the pot experiment. Plants were grown in two light conditions (SL: shade from sowing to the V5 stage and from V5 to R8 under natural light condition; LS: from sowing to the V5 stage under natural light condition and shade from V5 to R8) and two P application treatments (P0 = without P application; P100 = 100 mg P kg^−1^soil application). Values represent the mean of three replicates ± SE. Different letters on the columns are used to indicate means that differ significantly by LSD (*p* ≤ 0.05). For *GmYUCCA14* and *GmTIR1C* expressions, data are expressed as relative values based on the expression of *GmYUCCA14* and *GmTIR1C* in roots of soybean grown under the treatments of SL-P100 and SL-P100 at V5 stage referenced as 1.0, respectively.

At the V5 stage, the root IAA concentration in LS was significantly (*p* ≤ 0.05) higher than SL irrespective of P application (Figure 8A). On the contrary, the root IAA concentration in SL treatment was significantly (*p* ≤ 0.05) higher than LS at the R3 stage (Figure 8B). Whether it was the V5 or R3 stage, our results showed that auxin concentration in roots was increased by zero-P supply (Figure 8A,B).

Aboveground light conditions and P application had significant (*p* ≤ 0.05) effects on the expression of genes related to auxin synthesis and root response. *GmYUCCA14* and *GmTIR1C* were found to be predominantly expressed in soybean roots, implying their specific roles in root development [45,46]. In the present experiment, the expression of the gene *GmYUCCA14* encoding the auxin synthesis in soybean roots was increased by reducing the light intensity (Figure 8C). The *GmTIR1C* (auxin receptor) was highly expressed by reducing the light only under P deficiency at the V5 stage (Figure 8E). On the contrary, the expression of these two genes increased when the P supply was reduced and more light capture stimulated the expression at the R3 stage (Figure 8D,F).

## 3. Discussion

### 3.1. Root Growth and Distribution in the Variable Light Condition of Inter-cropped soybeans

Generally, crops acquire nutrients directly from soil, not water, in which the bioavailability of nutrients is typically suboptimal, especially the P nutrient [47]. Some important root architecture traits—such as total root length, root hair formation, and root branching—can be modified by P deficiency in order to enhance the P uptake capacity in the root system [48]. Consistent with those under P deficiency, plants under high light intensity showed a higher root/shoot ratio associated with greater carbohydrate concentrations in roots [18,49,50,51,52]. Inter-cropped soybeans suffered serious shade stress (low light intensity and quality) caused by maize during the common-growth period [18,53,54]. In the present study, we observed that variable light conditions in the relay strip inter-cropping system regulated soybean root growth and distribution. Soybean root growth in SL in the pot experiment was inhibited by shading at the V5 stage, as well as in the root biomass, total root length, and root surface area, all of which were significantly lower than LS treatment (Figure 3A,B). Harvesting empty strip maize leaves empty in the field increased light and growing space availability for inter-cropped soybeans, while the major leaves of monocropped soybeans were in a weak light condition because of the self-shading at the R3 stage [18,39]. Coincidentally, the length and surface area of lateral roots in SL were significantly higher, as compared to those in LS at the R3 stage (Figure 3D,F). However, the length and surface area of primary roots in SL were still lower than in LS (Figure 3C,E). The shade during the common-growth period inhibited not only the elongation of the primary root, but the formation and growth of the lateral root. Moreover, the inhibition of root elongation mainly affected cell differentiation and caused irreversible damage. These results indicated that the variable light (from shade to ameliorated light) of soybeans favored the formation of lateral roots rather than the growth of primary roots during the reproductive stage. This was considered to be the peak period of P uptake.

**Figure 9:**
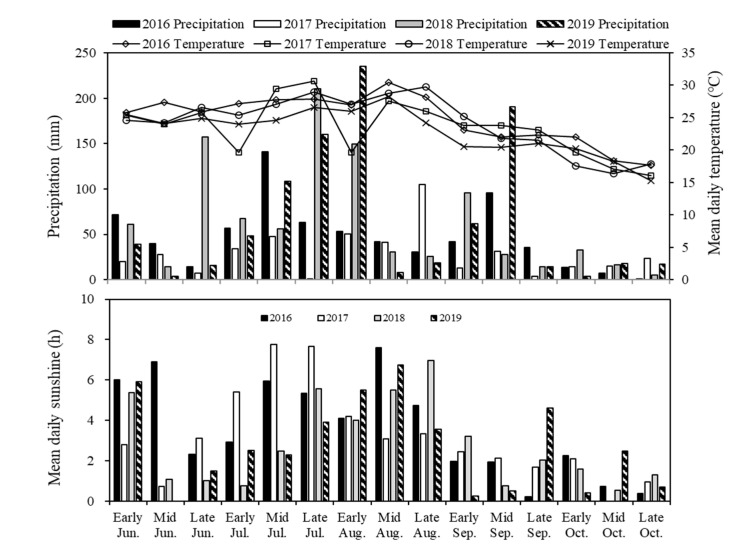
The 10-day precipitation, mean daily temperature, and sunshine hours during soybean growth season in 2016, 2017, 2018, and 2019.

P bioavailability is usually greatest in the topsoil because of P deposition from crop residues, limited P leaching to deeper soil strata, and greater biotic activity in the topsoil [55]. One opportunity to increase P uptake for crops is to improve foraging in the P-rich soil area (i.e., the topsoil in most agricultural soils). Topsoil foraging can be improved through greater lateral root density, greater root hair length/density, shallower root distribution, reduced root metabolic cost, and so on [47,55]. Except for root growth, the light condition of the inter-cropping system was also involved in distributing soybean roots in the soil profile. During the common-growth period, both the growth of primary and lateral roots were inhibited. At the R3 stage, more than 90% of the roots of inter-cropped soybeans were distributed in the top layer of 0–15 cm soil, whereas the distribution depth of vertical inter-cropped soybeans was shallower than for monocropped soybeans (Figure 2). Stout primary roost with a low density of lateral roots were found in monocropped soybeans, while a large number of secondary and tertiary lateral roots were observed in inter-cropped soybeans at the R3 stage (Figure S2C,D). The root architecture of inter-cropped soybeans was P efficient, but did not adapt to moisture stress. Fortunately, the precipitation from June to October (soybean growing season) accounts for more than 75% of precipitation for the whole year in this study area and so soybean growth is not subject to drought stress (Figure 9).

### 3.2. Auxin Concentration and Related Gene Expression Involves in Root Architecture

Our previous study proved that the formation of P-efficient root morphology at the R3 stage was attributed to the ameliorated light condition [18]. However, stimulating the lateral root formation increased light capture and was not exclusively controlled by an increased translocation of sucrose to roots. Instead, other systemic signals such as auxin could contribute to the induction of lateral root formation and growth [31,32,56,57,58].

To address the question of auxin’s role in the morphogenetic changes induced by the light condition, we analyzed the effect of auxin on root architecture and lateral root formation in different light environments. The results in the pot experiment showed that the IAA concentration in soybean roots was significantly decreased by the shade during the seedling stage, while the expression of the auxin synthesis gene *GmYUCCA14* and auxin receptor gene *GmTIR1C* were up-regulated in roots (Figure 8A,C,E). Our result is consistent with previous studies, which have indicated that continuous shade decreases photosynthesis, auxin synthesis substrates, and auxin concentration reduction, especially in the root [59,60]. Interestingly, in order to maintain Arabidopsis thaliana growth, the expression of receptor genes *AtTIR1*, *AtIAA19,* and *AtIAA29* that control the auxin response is significantly up-regulated in the continuous shade condition [59,60,61,62]. The results proved that the continuous shade can up-regulate the signal pathway involved in root auxin synthesis and response to maintain soybean root growth in the case of auxin concentration, which limits root growth. However, the expression of *GmYUCCA14* and *GmTIR1C* at the R3 stage were up-regulated by increased light intensity, which seems contradictory to the results (Figure 8D,F). In fact, this consequence is related to the growth character of soybeans. During the seedling stage, the root growth is regulated by photosynthates and nutrients acquired from soil by roots are supplied for shoot morphogenesis. However, nutrients acquired by roots during the reproductive period are mainly for grain formation and root growth enters a rapid growth period mainly regulated by shoot demand [63]. At the V5 stage, the photosynthetic capacity of soybeans in SL was limited by low light (Figure 5A) and root growth was inhibited by photosynthate. Thus, IAA synthesis and accumulation decreased in roots (Figure 8A). At the R3 stage, due to the weak light environment of soybeans in LS, the shoot demand decreased after sharply dropping in the flower and pod. Subsequently, the lateral root growth was slow (auxin regulation pathway was weakened). In contrast, both the IAA concentration and gene expression (*GmYUCCA14* and *GmTIR1C*) in SL at the R3 stage were up-regulated, which indicated that the shoot demand of soybeans dominated the lateral root formation (Figure 8B,D,F).

Except for shade, sucrose and P-deficiency up-regulated the sensitivity of auxin receptors [31,34,48,61,62]. Under low P conditions, IAA accumulation in the root tip inhibited the elongation of plant roots. After the sensitivity of the auxin receptor in mature regions increased, lateral root density was promoted [57]. In the present study, the P concentration of soybean leaves in SL at R3 stage was significantly lower than LS and the expression of the gene *GmPHR25* was also down-regulated (Figure 7B,D). Moreover, the sucrose concentration increased due to the ameliorated light condition (Figure 6B,D). The expression of the auxin receptor gene *GmTIR1C* in the root was up-regulated by the low P in leaves and high sucrose in roots. The density and length of lateral roots in SL were higher than soybeans in LS, especially in P0 treatment. At the R3 stage, the low P status in the leaves of SL increased the sensitivity of the auxin receptor gene and the lateral root length also increased, which seemed contrary to our previous study. The effect of P-deficiency on root elongation was mainly inhibited by the mitosis of cells in the meristematic zone so as to reduce the number and elongation growth of cells [64,65,66]. In the present study, low P concentration of leaves in SL during the R3 stage did not cause stress on plant growth. Moreover, the photosynthate produced in the shoot was translocated to the root as sucrose (Figure 6B,D), which served as both a signal for the integration of shoot light and P status that transduced to the root and a C-substrate for root growth [34,52,67,68]. Our results demonstrated that the increase of translocation in sucrose from shoot to root not only enhanced auxin synthesis but up-regulated the expression of the auxin receptor gene (Figure 8F). The light environment of SL at the R3 stage mediated sucrose and low-P signal to regulate auxin synthesis and sensitivity in the root, thus promoting the formation and elongation of the lateral root. A previous study in *Nicotiana tabacum* found that increased light intensity rapidly enhanced root growth by elevating sucrose export from shoot to root [28]. In addition, a similar result was found in white lupin, which increased the light mediating sucrose and auxin pathway resulted in greater production of cluster roots, and subsequently improved P uptake [52].

Root architecture, defined as the spatial configuration of a root system, is especially important for P acquisition by plants, since the relative immobility of P in soils makes P acquisition very dependent on soil exploration in time and space [69]. The results of the experiment reported here showed that root growth was regulated by auxin synthesis and response. However, it cannot completely explain the shallower root formation of the inter-cropped soybeans, because most of the lateral root arose at the 0–15 cm soil layer rather than the whole root. The two main reasons for the shallower root distribution of inter-cropped soybeans were as follows: (i) the ameliorated light significantly decreased the P concentration and up-regulated the expression of the low-P response gene (*GmPHR25*) in soybean leaves (Figure 7B,D). Meanwhile, the expression of *GmEXPB2* (encoding root growth) was also up-regulated (Figure 4B). P-deficiency inhibited the secondary growth of crop roots in dicots and legume crops with greater inhibition of secondary growth under P stress had reduced root costs and greater elongation growth [47,70]. The decrease of secondary growth showed that the elongation of the primary root was inhibited and the density of the lateral root significantly increased [71]. (ii) The inhibition of primary root growth was caused by shading during the early stage, which played a critical role in distributing lateral roots formed during the reproductive growth period of inter-cropped soybeans. The inhibition of the primary root modified the auxin synthesis center, transport pathway, and sensitivity of the auxin receptor gene located in the mature region in the root, thus promoting lateral root formation [48]. The growth of the primary root of soybean in SL was strongly inhibited with the destruction of the root tip and the auxin synthesis center was replaced by the primary lateral root tip at the V5 stage. Multiple primary lateral roots (instead of one primary root) provided more sites for secondary and tertiary lateral roots, ensuring the possibility of forming more lateral roots.

### 3.3. P Uptake Was Improved by the Relay Strip Inter-Cropping System

In the present study, inter-cropped soybeans had a P uptake advantage compared with monocropped soybeans across all 4 years (Figure 1C). The growth and development of lateral roots were crucial for maximizing the ability of the root to absorb P from the soil. Lateral roots assisted the acquisition of P by increasing the absorptive surface of the root and the formation of a highly branched root system may be a result of the canalization of carbon and energy resources to form a root system capable of exploring large areas of the upper soil layer, where P-rich patches are normally present [72]. The shade of inter-cropped soybeans suffered during the seedling stage and were inhibited by root the growth, especially the primary root. The ameliorated light condition after maize harvest mediation P status and photosynthate (sucrose) regulated the formation and growth of lateral roots, which were mainly distributed in the 0–15 cm soil profile. The early shading inhibited the development of the primary root and changed the auxin synthesis center from a primary root tip to multiple lateral root tips, which provided more sites for the occurrence of secondary lateral roots. During the reproductivity stage, the low-P signal and photosynthates formed by the ameliorated light not only increased the sensitivity to auxin but increased the IAA concentration in the root, thus increasing the density and length of the lateral root. Consequently, a root architecture with P-efficient uptake during the reproductive growth period was observed in the inter-cropped soybean. The P capture of inter-cropped soybeans was significantly improved because of the greater production of lateral roots, especially the hypocotyl-borne roots (Figure S2D), which typically had very shallow growth angles. Thus, the metabolic cost decreased [73,74].

In addition to the root architecture, the requirement for P in the shoot was a critical factor for the P uptake in soybeans. Available evidence suggests that the formation and nutrient uptake of roots are mainly regulated by shoot nutrient status through root split and local fertilization experiments [75,76,77,78]. The three major routes of shoot P status that regulate root growth and P uptake are as follows: (1) increased photosynthate distributes roots [51]; (2) systematic low P signal upregulates the expression of genes related to root growth [76]; (3) shoot P status mediates the synthesis and transport of hormones, such as promoting auxin transport from shoot to root, and increasing the sensitivity of auxin receptors in roots to increase lateral root density [48]. Due to the rapid growth rate during the vegetative growth period, monocropped soybeans suffered serious self-shading during the reproductive growth stage, which affected the photosynthetic capacity of the leaves located in the lower position. Moreover, flowers and pods decreased nutrient demand [18]. In contrast, the ameliorated light condition of inter-cropped soybeans significantly increased the photosynthetic capacity of the whole soybean plant and subsequently increased the requirement of P (Figure 5C). The findings of our study were consistent with the observations that inter-cropped soybeans have significant advantages of shoot P and PUE compared with monocropped soybeans (Figure 1C,D). However, the results showed that the average PUE of inter-cropping was only about 14.2% across 4 years, which may be attributed to the fact that soybeans absorbed a large amount of P soil rather than P fertilizer. Except for sucrose and auxin, other systemic signals could contribute to root growth. For example, ethylene was also involved in lateral root formation and primary root growth. Therefore, further work is needed to elucidate the role of ethylene when regulating the root growth of soybean in the variable light condition.

## 4. Materials and Methods

### 4.1. Field Experiment

#### 4.1.1. Site Description

Field experiments were carried out from 2016–2019 at the department of Sichuan Agricultural University Renshou Experimental Station (30°06′ N, 104°02′ E) in Sichuan Province, China. Annual mean temperature was 17.4 °C with a maximum and minimum temperature of 25.4 °C and 6.1 °C, respectively. Annual precipitation was 1009.4 mm, annual sunshine was about 1196.4 h, and total solar radiation averages was around 3850 MJ m^−2^ yr ^−1^. The detailed meteorological data during the during soybean growth season from 2016 to 2019 are shown in Figure 9. The experimental soil was classified as purple soil (Luvic Xerosols) and Olsen-P content was low. The soil characters at the study site in 2016 were as follows: pH (1:2.5, soil:water) 6.8, cation exchange capacity 22.1 cmol kg^−1^, organic matter content 20.5 g kg^−1^, total nitrogen (N) 1.5 g kg^−1^, available N 0.11g kg^−1^, exchange K 0.115 g kg^−1^, and Olsen-P 12.6 mg kg^−1^ of dry soil in the top 20 cm soil layer. After the present study, the available P content in the treatment P0 and P20 were 8.5 mg kg^−1^ and 14.6 mg kg^−1^, respectively.

#### 4.1.2. Experiment Design and Crop Management

The field experiment was a split-plot design. The main plot included two P application rates (P0: zero-P supply and P20: 20 kg P ha^−1^) and the sub-plot consisted of a maize (*Zea mays* L. cv. Chuandan no.418)/soybean (*Glycine max* L. cv. Nandou No. 12) relay strip in an inter-cropped soybean system (*Glycine max* L. cv. Nandou No. 12) and monocropped soybean system. Chuandan No. 418 is one of the 10 main popularized maize varieties in Sichuan Province. It is a semi-compact maize variety suitable for planting in the maize-soybean relay strip. Nandou No. 12 is a Sichuaneses summer soybean cultivar with the largest acreage. It is grown wildly in the maize-soybean relay strip inter-cropped system because of its shade-tolerant. The area of each individual plot was 6.0 × 5.0 m^2^ for inter-cropping and 3.5 × 5.0 m^2^ for monocropping. Each inter-cropped plot consisted of 3 strips with 2.0 m in width (distance between maize rows was 0.4 m and gap width for growing soybeans was 1.6 m) and the maize/soybean row ratio was 2:2, while each monocropping plot consisted of 7 soybean rows (distance between rows was 0.5 m). The density of inter-cropped maize was 60,000 plants per hectare and the density of both inter-cropped and monocropped soybeans were 105,000 plants per hectare.

The sowing dates of maize from 2016 to 2019 were on 3rd April, 4th April, 2nd April, and 30th March; harvest dates were on 30th July, 30th July, 1st August, and 28th July of that year. Soybean sowing dates were on 20th June, 20th June, 15th June, and 21st June; harvest dates were on 30th October, 30th October, 2nd November, and 29th October of that year. The seedling phase of the soybean and reproductive phase of maize overlapped over a period of approximately 40 days between soybean sowing and maize harvesting.

All of the urea (30 kg N ha^−1^), superphosphate, and potassium chloride (20 kg K ha^−1^) fertilizers were applied as basal fertilizer before soybean sowing. The fertilization of inter-cropped soybeans was located on the side of the wide row. The distance from the seeds was 3–5 cm and the depth was 5 cm, while the fertilization position of the monocropped soybeans was at the left below the seed (to avoid damage to young roots caused by fertilizer). During the whole process of the experiment, no application of organic fertilizer or artificial irrigation were used. Weeds were removed manually during the whole growth period and pesticide control were used in flowering stage of soybean.

#### 4.1.3. Plant Harvest and Measurements of Yield and P Concentration.

From 2016–2018, samples at the maturity stage were collected. In each plot, 5 plants with uniform growth were cut off from the base of the stem, which were divided into stem and pod, and subsequently dried at 75 °C to calculate the biomass. To measure the grain yield, all the middle strips of soybeans in each plot were harvested and measured the effective plant number, then naturally air-dried, and finally the actual grain yield was calculated. The samples at the stages of V5 (Five trifoliolates unroll), R3 (Pods are about 5 mm long on one of the four uppermost nodes on the main stem with a fully developed leaf), and R8 (95% of pods reach mature pod color) were collected in 2019. The sampling method at the stage of R8 was the same as mentioned in maturity. Next, 5 plants with uniform growth in each plot were cut off from the base of the stem, which were divided into stem and leaf, then dried at 75 °C to calculate the biomass at the stages of V5 and R3. The dry material was ground to pass through a 0.149-mm mesh sieve, then a 0.3-g sample was digested with concentrated H_2_SO_4_ and H_2_O_2_ (30% *v*/*v*), and P was determined by the vanadomolybdate method [79].

#### 4.1.4. Determination of Root Distribution

In order to obtain the biomass of the soybean root system and its distribution in soil, layer by layer and blocks extraction were adopted to dig out the roots from the soil in 2018. The volume of each small soil block of inter-cropped soybeans was about 20 cm (length) × 10 cm (width) × 15 cm (height), and the total volume of dug soil was 110 cm (row spacing) × 20 cm (nest spacing) × 60 cm (depth). The volume of each monocropped soybean was 20 cm (length) × 10 cm (width) × 15 cm (height), and the total volume of soil dug was 50 cm (row spacing) × 20 cm (nest spacing) × 60 cm (depth). The roots were carefully picked out from the soil, and washed free of soil then dried at 75 °C to calculate biomass.

### 4.2. Pot Experiment

#### 4.2.1. Site Description

In 2019, the pot experiments were conducted in the Teaching Farm of Sichuan Agricultural University (30°42′ N, 103°51′ E), which is located in Wenjiang District, Chengdu City, Sichuan Province. The field and pot experimental site were 150 km apart and had similar climatic conditions, such as precipitation, sunshine hours, temperature, and so on. The paddy soil (Ochric Aquic Cambosol soil) was used in the pot experiment and the soil characters were as follows: pH (1:2.5, soil:water) 6.7, organic matter content 8.3 g kg^−1^, total N 0.21 g kg^−1^, available N 0.1g kg^−1^, exchange K 0.103 g kg^−1^, and Olsen-P 3.0 mg kg^−1^.

#### 4.2.2. Experiment Design and Crop Management

In the pot experiment, soybean (*Glycine max* L. cv. Nandou no.12) was grown in shade-light (SL) and light-shade (LS) with two P application rates (P0: zero-P supply; P100: 100 mg P kg^−1^ soil). The sunshade net was used to simulate the light environment in the field. The light transmittance of sunshade net measured by HR350 (Hipoint Inc., Gaoxiong, Taiwan) was about 50%. Shading the whole soybean plant from sowing to V5 then lighting was used to imitate the light condition of soybean in the relay trip inter-cropping system, while soybean shaded all around from V5 to maturity was used to imitate the light condition of monocropped soybeans. The volume of the pot was about 0.021 m^3^ and each pot was filled with 10 kg air-dried soil. To ensure that the nutrient supply was sufficient for soybean growth, soil was fertilized with basal nutrients at the following rates (pot^−1^): Ca(NO_3_)_2_·4H_2_O 16,870 mg, CaCl_2_ 1260 mg, MgSO_4_·7H_2_O 430 mg, ZnSO_4_·7H_2_O 100 mg, MnSO_4_·4H_2_O 67 mg, EDTA-FeNa 58 mg, CuSO_4_·5H_2_O 20.0 mg, H_3_BO_3_ 6.7 mg, and (NH_4_)_6_Mo_7_O_24_·4H_2_O 2.6 mg. In the P100 treatment, KH_2_PO_4_ was used as P fertilizer and K_2_SO_4_ was used to make up the potassium in the P0 treatment. One month before sowing, all fertilizers were manually mixed into the soil, then water was added and placed until sowing. After aging, the content of available P in soil of P0 and P100 were 3.0 and 24.7 mg kg^−1^, respectively.

Before sowing, the same sized seeds with intact seed coats were selected and sterilized with 30% *v/v* H_2_O_2_ for 20 min. There were 20 pots in each treatment and 4 seeds were sowed. In total, 2 plants in each pot were kept for 5 days after emergence. Each pot was covered with foam cardboard to prevent rain from entering and all pots were watered daily to control the soil water content about 70% of the field capacity until harvest. The main sampling period were concentrated at the V5, R3, and R8 stages, and the specific dates were 31st July, 10th September, and 30th October, respectively.

#### 4.2.3. Determination of P, Net Photosynthesis, Sucrose and Auxin Concentration.

At the stages of V5, R3, and R8, the P content was determined in the shoot and root materials after digesting in a mixture of H_2_SO_4_ and H_2_O_2_ (30% *v*/*v*). P concentration was determined by the vanadomolybdate method [79].

Net photosynthesis (Pn) was measured at the middle part of the youngest expended leaf by using a Li6400 photosynthesis system (Li-COR, Lincoln, NE, USA). Measurement was conducted between 10:00 a.m. and 12:00 p.m. at the V5 and R3 stages.

Individual samples of leaves and roots were collected at about 18:00 in the afternoon of the sampling day, homogenized, and extracted with 80% ethanol. Sucrose was measured directly in the extract by the spectrophotometer DU-730 (Beck Man Coulter Inc., Brea, CA, USA), using resorcinol as the color reagent [80].

To measure the root auxin concentration, samples of root tips were collected. We removed the soil completely from the pot, put it into running water, and rinsed slowly until the root system was exposed, then washed it with deionized water. Finally, the 2 cm-long root tips were cut off with sterilized scissors and immediately preserved in liquid nitrogen, and then brought back to the laboratory for storage at −80 °C. Determination of IAA concentration in roots by an enzyme-linked immunosorbent assay (ELISA). The standard sample and antibody of auxin were provided by China Agricultural University.

#### 4.2.4. Measurements of Root Morphology

At the V5 and R3 stages, the root samples were washed with deionized water. Before scanning, the soybean root samples were divided into primary root and lateral roots. (The primary root referred to the primary root of biology and the attached fine root, while the lateral root referred to the branches growing from the primary root and all the fine roots on the branches.) Then, the root samples were scanned using an Epson Perfection V700 Photo scanner (Nagano-ken, Japan). Images were analyzed using WinRHIZO (WinRHIZO Pro2004, Quebec, Canada) to calculate the root length and surface area.

#### 4.2.5. Quantitative Real-Time PCR Analysis

Excised tissues (leaves and roots) were immediately frozen in liquid nitrogen and stored at −80 °C until gene expression analysis. Total RNA was extracted using a RNAisoTM Plus reagent (TaKaRa, Tokyo, Japan). The obtained RNA sample was treated with RNase-free DNase I, then MMLV-reverse transcriptase (Promega) was used to synthesize the first strand cDNA. Quantitative real time RT-PCR was performed using the SYBR Green PCR Master Mix (TaKaRa, Tokyo, Japan) in the QuantStudio 6 Flex real-time PCR detection system (Thermo Fisher Scientific, Waltham, MA, USA) with appropriate primers. Relative expression of genes compared to ACTIN was calculated using the ΔΔCt method [81].

The primers used to quantify gene expression were: *GmACTIN*, 5′-ATCTTGACTGAGCGTGGTTATTCC-3′ and 5′-GCTGGTCCTGGCTGTCTCC-3′; *GmPHR25*, 5′-AAAGGCCGACAAGAAAGAAACAGG-3′ and 5′-AACCACCGCTACAGCACCAGAAC-3′; *GmEXPB2*, 5′-TACCCTCCTCTTGTTTCAACCCT-3′ and 5′-AGCACCACCTTCACTACCGTCC-3′; *GmYUCCA14*, 5′-GATCTTCCATCCTTGGAGGC-3′ and 5-GCAACAGTATGCACTTGACCTTAC-3′; *GmTIR1C*, 5′-GGTGACAAGGCCCTTTTG-3′ and 5′-CTCACCGAGCAGGAGGAC-3′;

### 4.3. Statistical Analyses

The two-dimensional distribution of the root system in soil was simulated using the Surfer (Surfer 8.0, USA) software according to the root dry weight. The calculation of the root dry weight ratio of 0–15 cm soil layer (i.e., the proportion of root distributed in the upper 15 cm soil layer, RU15) was as follows:

(1) $$RU15=\frac{root\;dry\;weight\;in\;the\;upper\;15\;cm\;soil}{total\;root\;dry\;weight}\;\times 100\%$$

The formula for calculating the apparent P fertilizer-use efficiency (PUE) was as follows:

(2) $$\mathrm{PUE}\left(\%\right)=\left(\frac{U_{f}-U_{0}}{P_{f}}\right)\times 100$$

Among them, *Uf* represented the amount of P accumulation in the shoot of the P20 treatment, *U0* represented the amount of P accumulation in the shoot of the P0 treatment, and *Pf* referred to the total amount of P fertilizer application.

The relationship between the standard curve auxin concentration and the measured OD value was fitted by the Logit equation:

(3) $$\mathrm{Logit}\frac{B}{B_{0}}=\ln\frac{B/B_{0}}{1-B/B_{0}}=\ln\frac{B}{B_{0}-B}$$

Among them, B_0_ was chromogenic value of 0 ng mL^−1^, while B was the chromogenic value of other concentrations. According to the logit of the chromogenic value, the natural logarithm of the hormone concentration (ng mL^−1^) can be found out from the graph, and then the hormone concentration (ng mL^−1^) can be calculated by the inverse number. After obtaining the auxin concentration of the samples, the auxin content of soybean root (ng g·fw^−1^) could be calculated according to the volume fraction of the whole extraction and dilution process and the weight of the sample.

The data were sorted in the Excel 2019 (Microsoft) software package and then the significant differences among means were separated according to LSD at the level of *p* ≤ 0.05 using a SPSS statistical software package (Version 19.0, SPSS Institute Inc., New York, NY, USA). All data were subjected to one-way ANOVA to assess the significant differences among the four treatments. The tables and figures were made by Excel 2019 and Origin 2020b.

## 5. Conclusions

Here, our results demonstrated that the increase in lateral root formation and soybean growth in variable light environments are, at least in part, mediated by increases in auxin level and root sensitivity. The ameliorated light during the solo-existing period increased the IAA concentration in inter-cropped soybean roots. Moreover, the inhibition of plant growth caused by shade during the common-growth period leads to growth-induced P deficiency during the reproductive stage, and subsequently, upregulates the auxin response. It was envisaged that the variable light condition (shade-light) mediated leaf P status and sucrose to regulate auxin synthesis and response, which resulted in more lateral root growth and shallower root distribution of soybeans in the relay strip inter-cropping system. This study enriches the theory of increasing production and efficiency of strip inter-cropping system, providing a reference for taking strip inter-cropping as an effective agro-ecological technology to efficiently manage farmland P resources and to maintain soil fertility.

## Supplementary Materials

The following are available online at https://www.mdpi.com/2223-7747/9/9/1204/s1, Figure S1: The shoot P accumulation as affected by the P application and light condition in the pot experiment. Figure S2: Effect of cropping system on the root morphology and distribution; Table S1: The dry matter and shoot P accumulation (g m^−2^) of soybeans during the growth period as affected by P application and cropping system in the field experiment.
