# Supplementary material for: Variable Light Condition Improves Root Distribution Shallowness and P Uptake of Soybean in Maize/Soybean Relay Strip Intercropping System
Source: Plants (Basel). 2020 Sep 15;9(9):1204. doi: 10.3390/plants9091204 (PMC7570427; doi:10.3390/plants9091204)
Supplement: Supplementary file 1 [file plants-09-01204-s001.pdf]

Fig. S1

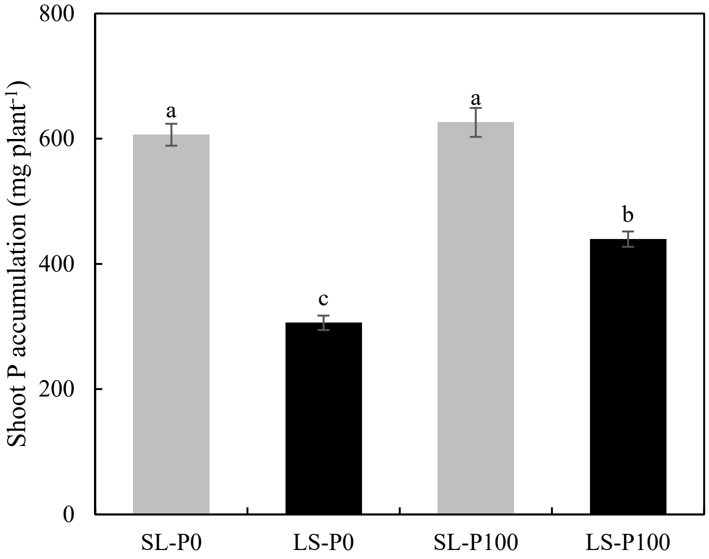

SFig. 1 The shoot P accumulation as affected by the P application and light condition in the pot experiment. Plants were grown in two light treatments (SL: planting to V5 stage under shade condition, V5-R8 in natural light condition; LS: planting to V5 stage under natural light condition, V5-R8 under shade condition) and two P application treatments (P0: with zero P application; P100: with 100 mg P kg^{-1}soil application). Data are averages of three replicates and bars represent standard errors. Data with different letters are significantly different by LSD ($p \le 0.05$).

Fig. S2

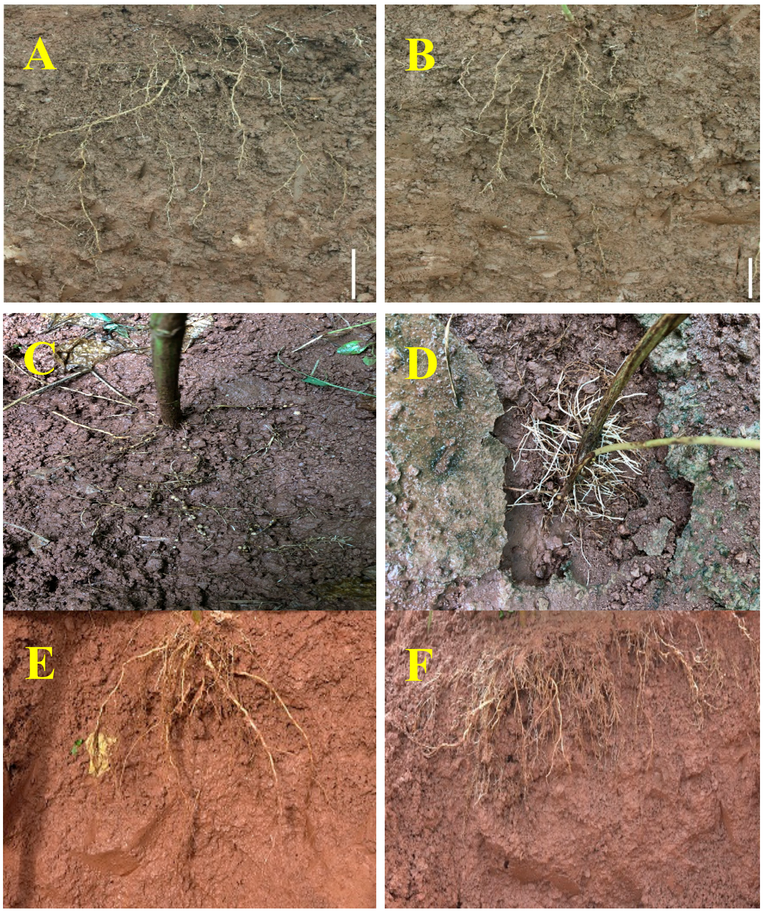

Fig. S2 Effect of cropping system on the root morphology and distribution (A: monocropped soybean at V5 stage; B: intercropped soybean at V5 stage; C: monocropped soybean root in the topsoil at R3 stage; D: intercropped soybean root in the topsoil at R3 stage; E: monocropped soybean root belowground at R3 stage; F: intercropped soybean root belowground at R3 stage;) in the field experiment.

Table S1 The dry matter and shoot P accumulation (g m^{-2}) of soybean during the growth period as affected by P application and cropping system in the field experiment.

Measurements	P rate	V5		R3		R3/V5	
		Inter.	Mono.	Inter.	Mono.	Inter.	Mono.
Dry matter	P0	13.3 c	34.8 b	273.3 d	428.1 b	20.6 a	12.3 b
	P20	14.9 c	50.5 a	352.4 c	497.4 a	23.7 a	9.9 b
Shoot P	P0	0.022 d	0.058 b	0.563 c	0.843 b	26.1 a	14.5 b
	P20	0.039 c	0.113 a	0.891 b	0.983 a	23.1 a	8.7 c

Plants were grown in intercropping (Inter.) and monocropping (Mono.) systems with two P application rates (P0: with zero P supply; P20: with 20 kg P ha^{-1}supply). Data are average of three replicates. Values followed by the same lowercase letters are not significantly different between sole and intercropping cropping system within the same P rate in one year at the 5% level by LSD.
